# Diagnostic value of prenatal ultrasound in the typing of fetal esophageal atresia

**DOI:** 10.3389/fmed.2025.1595265

**Published:** 2025-07-29

**Authors:** Lijun Song, Zhijie Zhang, Xuan Sheng, Yanjie Wang, Houmei Han, Yang Gao, Hong Yin

**Affiliations:** Department of Ultrasound, Shandong Maternal and Child Health Hospital, Jinan, China

**Keywords:** esophageal atresia, ultrasound examination, prenatal diagnosis, classification, congenital malformations

## Abstract

**Objective:**

To investigate the prenatal ultrasound image features and diagnostic value of fetal esophageal atresia (EA).

**Methods:**

The clinical and prenatal ultrasound data of 24 fetuses with suspected esophageal atresia diagnosed by prenatal ultrasound and/or postpartum ultrasound at Shandong Maternal and Child Health Hospital between February 2022 and November 2024 were retrospectively analyzed, and the prenatal ultrasound diagnostic characteristics of different types of esophageal atresia were reviewed retrospectively.

**Results:**

Twenty-four cases of esophageal atresia were detected by prenatal and/or postpartum ultrasound. Among the 11 patients who were lost to follow-up or with no clear diagnosis after birth, the confirmation rate with prenatal ultrasound was 92% (12) among the 13 cases with confirmation by postpartum surgery or anatomy, with a classification concordance rate of 77% (10/13). Two cases of type IIIA EA were misdiagnosed as type IIIB, and one case of type IIIB EA was missed. The proportion of other malformations was 77% (10/13), and the detection rate of chromosomal abnormalities was 15% (2/13).

**Conclusion:**

Prenatal ultrasound can be used to diagnose and assess the classification and combined malformations of esophageal atresia effectively, providing an strong basis for clinical decision-making.

## 1 Introduction

Esophageal atresia (EA) is a severe congenital malformation caused by incomplete foregut separation or esophageal cavitation disorders in the early embryo, and the incidence rate is approximately 1/2,500–3,800 fetuses (1). Prenatal ultrasound remains the gold standard for detecting fetal structural anomalies, including gastrointestinal malformations such as anal atresia and esophageal atresia (2, 3). Recent studies confirm its diagnostic superiority over MRI and amniotic fluid biochemistry (e.g., GTPP/AFP) due to cost, accessibility, and dynamic assessment capabilities (1, 4, 5). For EA specifically, advancements in high-resolution ultrasound have enabled direct visualization of tracheoesophageal fistulas and blind-end distances, refining prenatal classification (6–9). In this study, the ultrasound features of 24 suspected cases of EA were analyzed, and the results of postpartum autopsy or surgery were used as the gold standard to explore the diagnostic value of prenatal ultrasound for different types of EA.

## 2 Materials and methods

### 2.1 Research objects

A total of 24 EA fetuses diagnosed by antenatal/postpartum ultrasound in our hospital between February 2022 and November 2024 were included and approved by the Ethics Committee of Shandong Maternal and Child Health Hospital affiliated with Qingdao University (ethics number: NO. 2018-039).

### 2.2 Instruments and methods

Using a GE Voluson E10 ultrasound system (C1–6D probe, 1.0–5.0 MHz), the indirect signs were focused on the amniotic fluid volume (AFI), the size of the gastric blebs, and the direct signs: the esophageal pouch sign, the interruption sign, and the esophagotracheal fistula.

The classification of esophageal atresia is as follows (10):

Type I: simple esophageal atresia.

Type II: esophageal atresia with upper esophagus-tracheal fistula.

Type III: esophageal atresia with a lower esophageal-tracheal fistula (IIIA: distance between the two blind ends of the esophagus ≥2 cm; IIIB: distance between the blind ends of the esophagus <2 cm).

Type IV: esophageal atresia with upper and lower esophageal-tracheal fistulas.

Type V: no esophageal atresia, simple esophagotracheal fistula.

Ultrasound examination methods of: the pregnant woman was placed in the supine position, and the fetus was subjected to prenatal ultrasound examination via the abdomen. When fetal amniotic fluid and small gastric bubbles were found, the esophagus and trachea were continuously observed via the parasternal longitudinal view, the paraaortic longitudinal view, and the esophagus and trachea short-axis view, and the esophagus was repeatedly and continuously explored for discontinuity signs and pouch signs. The presence of esophagotracheal fistula was observed, and the presence of other malformations was screened in detail.

## 3 Results

### 3.1 Basic information

**Figure 1:**
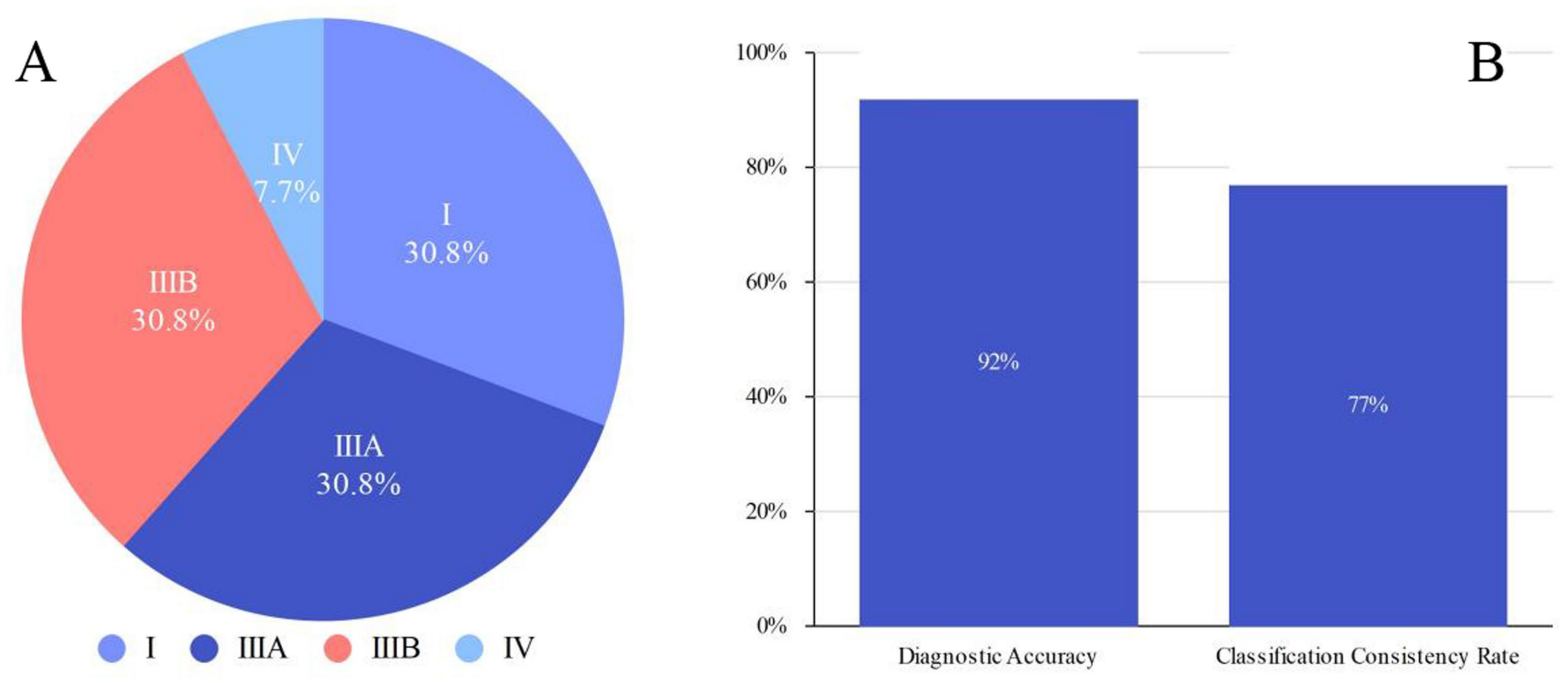
**(A)** Distribution of EA types (I, IIIA, IIIB, IV) in confirmed cases. **(B)** Diagnostic accuracy and classification consistency rate in confirmed cases.

**Figure 2:**
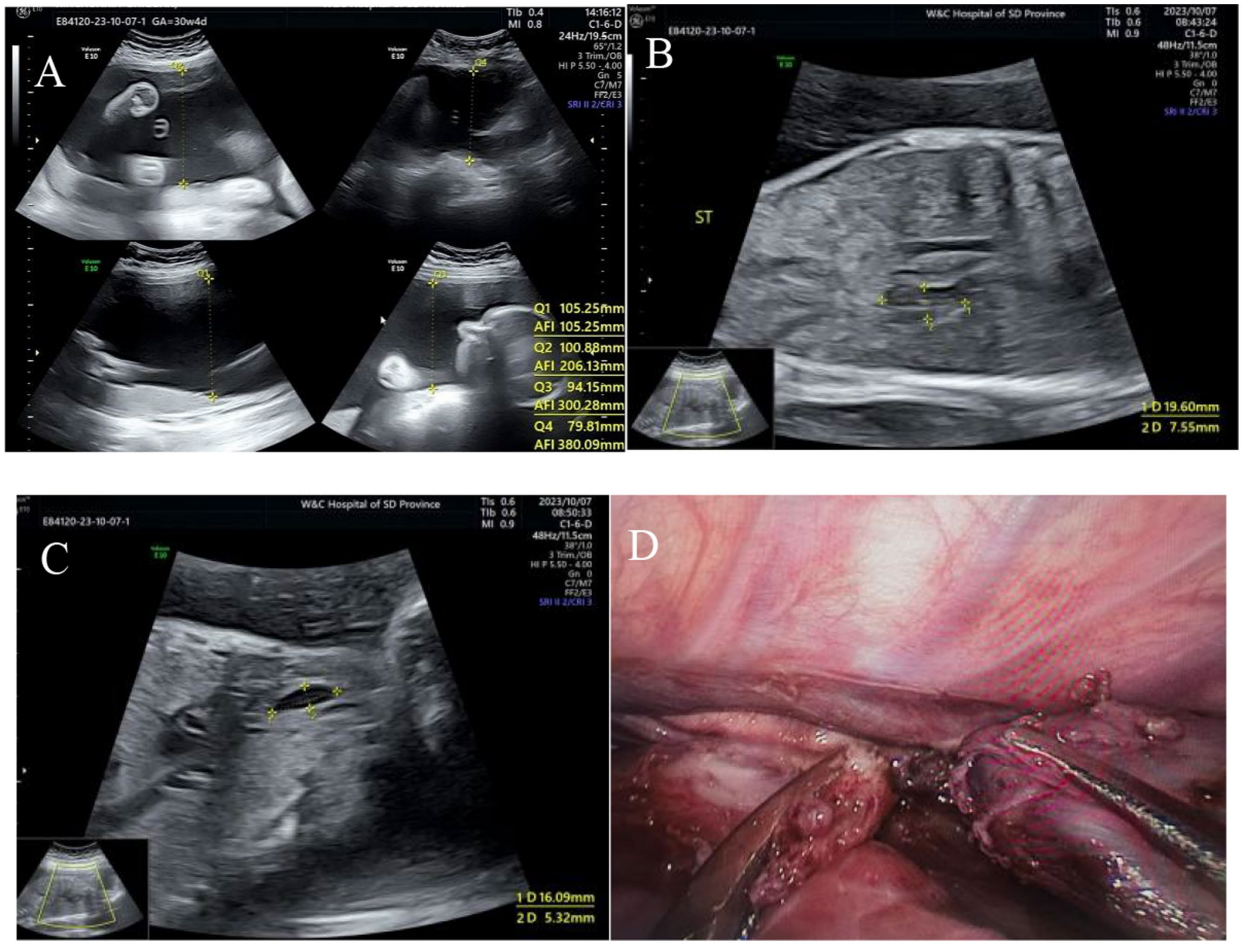
Type I esophageal atresia. **(A)** Polyhydramnios fluid; **(B)** small gastric vesicles; **(C)** pouch sign in the upper esophagus; **(D)** surgical photograph.

**Figure 3:**
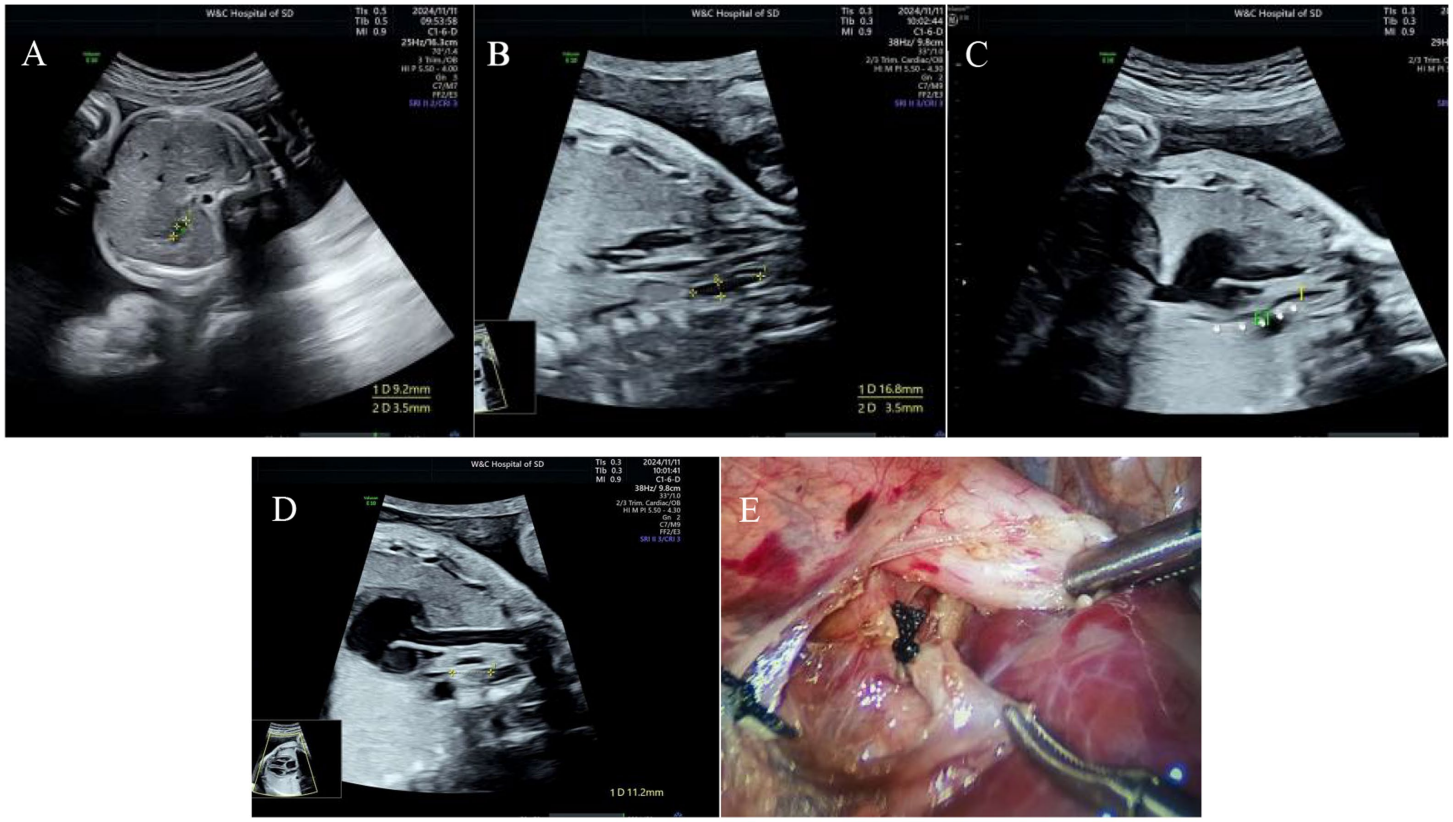
Type IIIB esophageal atresia. **(A)** Small gastric bubble; **(B)** pouch sign in the upper esophagus; **(C)** esophagus-tracheal fistula in the lower segment; **(D)** the distance between the two blind ends of esophagus was approximately 10 mm; **(E)** surgical photograph.

**Figure 4:**
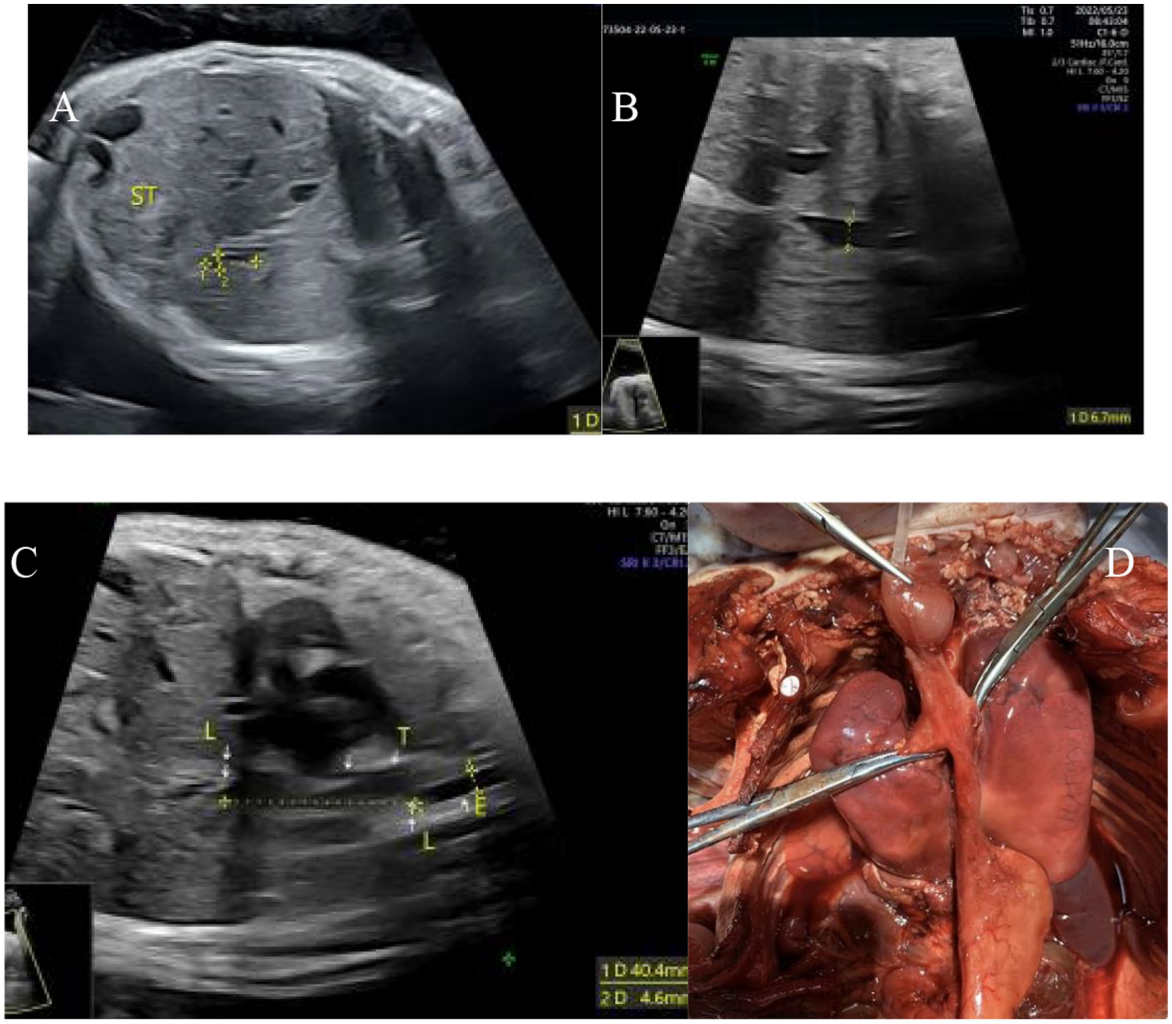
Type IV esophageal atresia. **(A)** Small gastric bubble; **(B)** upper esophagus interruption sign; **(C)** esophagus-tracheal fistula in the upper and lower segments; **(D)** autopsy image.

Among the 13 fetuses with confirmed EA, 30.8% (4/13) had type I EA, 30.8% had type IIIA EA (4/13), 30.8% had type IIIB EA (4/13), and 7.7% had type IV EA (1/13). There were 5 male fetuses and 7 female fetuses. There were 2 cases of SARS-CoV-2 infection in the first trimester and 2 cases of chromosomal abnormality (trisomy 18). A total of 12 patients were diagnosed with EA by prenatal ultrasound, with a diagnostic accuracy of 92% (12/13). Ten cases were correctly classified, with a classification consistency rate of 77% (10/13) (see Figure 1). Two patients with type IIIA EA were misdiagnosed as having type IIIB EA. One case was missed and confirmed by postpartum surgery to be a case of type IIIB EA (see Table 1 and Figures 2–4).

**Table 1:** Prenatal ultrasound manifestations and follow-up results of EA.

Cases	Age (years)	Gestational age (week)	Ultrasound manifestations					Ultrasound classification	Pregnancy outcomes	Birth weight (g)	Gender	Chromosomal examination	Postmortem autopsy or surgical diagnosis
			AFI/AF* (mm)	Small/no gastric bubbles	Upper esophagus signs	Esophagotracheal fistula	Combined with other abnormalities						
1	30	24	155	/	/	/	/	/	Normal delivery	3,030	M	Not done	Type IIIB
2	31	30	383	Small	Pouch sign	Lower paragraph	Dextral heart	Type IIIA	Cesarean section	2,250	M	Normal	Type IIIA
3	26	37	454	None	Pouch sign	Lower paragraph	/	Type IIIB	Cesarean section	2,530	M	Normal	Type IIIA
4	36	27	228	None	Pouch sign	/	Ventricular septal defect, FGR Double inferior vena cava, single umbilical artery	Type I	Cesarean section	1,740	F	Normal	Type I
5	31	26	380	Small	Pouch sign	/	FGR	Type I	Cesarean section	2,450	F	Normal	Type I
6	34	24	288	Small	Pouch sign	Lower paragraph	Anal atresia, ventricular septal defect Right umbilical vein	Type IIIB	Cesarean section	2,540	F	Normal	Type IIIA
7	30	35	280	Small	Discontinuation sign	Lower paragraph	FGR	Type IIIB	Cesarean section	2,110	M	Normal	Type IIIB
8	30	24	405	None	Pouch sign	/	Double superior vena cava, right subclavian artery	Type I	Cesarean section	2,490	M	Normal	Type I
9	31	23	310	Small	Discontinuation sign	Lower paragraph	Complete ectopic pulmonary venous drainage Hemi vertebral body VACTERL	Type IIIB	Induction of labor	2,263	M	Trisomy 18	Type IIIB
10	32	34	298	None	Discontinuation sign	Lower paragraph	FGR	Type IIIB	Cesarean section	2,415	F	Normal	Type IIIB
11	42	35	300	Small	Discontinuation sign	Upper and lower paragraphs	Ventricular septal defect, double superior vena cava Portosystemic shunt, single umbilical artery Absence of venous catheter	Type IV	Induction of labor	2,510	M	Trisomy 18	Type IV
12	29	24	118*	Small	Pouch sign	Lower paragraph	/	Type IIIB	Cesarean section	2,850	F	Normal	Type IIIA
13	38	36	285	/	Pouch sign	/	Duodenal atresia, anal atresia	/	Cesarean section	2,515	M	Normal	Type I

AFI, amniotic fluid index; AF*, amniotic fluid.

### 3.2 Ultrasound features

Among the 13 fetuses with confirmed EA, 11 had polyhydramnios, and 6 had an amniotic fluid index >300 mm in the third trimester of pregnancy. Gastric blebs were not present in 4 patients, persistent small gastric blebs were present in 7 patients, the size of the gastric blebs was normal in 1 patient, and the gastric blebs were dilated in 1 patient. There were 8 patients with esophageal bag signs, 4 patients with esophageal interruption signs, 0 patients with upper esophagus-tracheal fistulas, and 8 patients with lower esophagus-tracheal fistulas.

### 3.3 Coalescing exceptions

Among the 13 fetuses with confirmed EA, 77% had other abnormalities (10/13), and 46% had multiple abnormalities (6/13), including VACTERL syndrome in 1 patient. Other abnormalities included growth restriction in 4 cases, ventricular septal defect in 3 cases, double superior vena cava in 3 cases, anal atresia in 2 cases, a single umbilical artery in 2 cases, complete pulmonary venous drainage in 1 case, a dextral heart in 1 case, and varicose veins in 1 case. There was 1 case of right subclavian artery involvement, 1 case of hemivertebra involvement, 1 case of portosystemic shunt involvement, and 1 case of absent venous catheter.

### 3.4 Amniocentesis

All 13 fetuses with confirmed EA underwent amniocentesis for karyotype analysis, and 2 fetuses had trisomy 18.

### 3.5 Prognosis

There was 1 neonate with a weight less than 2,000 g, 6 neonates with a weight ranging from 2,000 to 2,500 g, 5 neonates with a weight ranging from 2,500 to 3,000 g, and 1 neonate with a weight greater than 3,000 g. Among the procedures used in the 13 cases of confirmed EA, induction of labor was performed in 2 cases, treatment was provided after birth in 11 cases, good recovery occurred after birth in 9 cases, and treatment was discontinued due to multiple postoperative complications in 2 cases.

## 4 Discussion

Normally, at 3–5 weeks of gestation, the lung bud develops into the primordial foregut, which is subsequently dissects into the trachea and esophagus (11). Blockage of this process leads to abnormal dissection of the foregut and the development of esophageal atresia. One study (7) reported that the process of esophageal and tracheal differentiation and maturation is regulated by genes, especially growth factors (such as *NODAL* and FGF4) and transcription regulators (such as *Foxf1* and Sox2). The interruption of signaling pathways (such as the *Fgf Wnt* pathways) affects the normal expression of genes, resulting in the development of abnormal foregut dissection and esophageal atresia.

According to the diagnostic criteria of “Pediatric Surgery,” the commonly used classifications of esophageal atresia in China (10) are type I, type II, type III (IIIA, IIIB), type IV, and type V, which were used in the present study. The commonly used standard for prenatal ultrasound diagnosis of EA in other countries is the Gross and Vogt classification (6), but these methods do not involve measuring the distance between the two ends of the esophagus, which is therefore limited. The domestic standards detail types IIIA and IIIB, which are more important in clinical practice.

The incidence of esophageal atresia in fetuses varies according to the type of EA (12). In total, 4–8% of fetuses have type I EA, 1–2% of fetuses have, 85–90% of fetuses have type III EA, 1–5% of fetuses have type IV EA, and 4–6% of fetuses have type V EA (13). In this study, type III accounted for the greatest proportion of cases, followed by type I, which was basically consistent with the findings of previous studies. Literature reports (14) indicate that the incidence rate of EA is 3:2 for males and females. In this study, among the 13 confirmed fetal cases, 8 were male and 5 were female, which is in good agreement with previous reports. It has also been reported (15) that the incidence of EA is increased in first-time pregnancies, advanced diabetes in pregnancies, and twin pregnancies. In addition, infection during pregnancy and long-term use of estrogen or thalidomide also have teratogenic effects.

Currently, prenatal ultrasound is the preferred method for diagnosing fetal esophageal atresia. In recent years (3–5, 16, 17), MRI and amniotic fluid biochemistry, such as GTPP and AFP, has been reported to supplement the prenatal ultrasound findings in determining the diagnosis. However, the examination cost is high, the availability of MR images is poor, the quality of the MR images may be affected by fetal movement, and the biochemical specificity of amniotic fluid is low. Therefore, most medical units, even tertiary fetal medical centers, seldom use MRI and amniotic fluid analysis to supplement ultrasound screening routinely, and clinically, they rely mainly on prenatal ultrasound diagnosis (5). Therefore, it is particularly important to improve the diagnostic ability of prenatal ultrasound for esophageal atresia.

The common prenatal ultrasound signs of esophageal atresia are “polyhydramnios” and “small/nongastric bubbles.” The reason is that esophageal atresia prevents amniotic fluid from entering the fetus’s stomach normally, resulting in the accumulation of amniotic fluid in the mother’s body. The above two indirect signs have high diagnostic sensitivity but low specificity, especially when there is no abnormality in the amniotic fluid or gastric vesicles, so it is easy to miss the diagnosis (1, 3). The positive predictive rate of the absence of a gastric bleb combined with polyhydramnios for fetal esophageal atresia is only 42–56% (18). These two signs have a relatively high prediction rate for type I and type III esophageal atresia. Because there is no fistula opening between the esophagus and trachea in the two types of upper segment atresia, the display rate of these two signs is 85% (19). Among the 13 fetuses diagnosed in this study, 11 had small/nongastric bubbles, 11 had polyhydramnios, and the highest AFI was 454 mm, which may be due to the greater proportion of type I and type III EA.

In recent years, the emergence of the “esophageal pouch sign” has improved the accuracy of prenatal ultrasound for the diagnosis of EA (18). When the fetus swallows amniotic fluid, dilatation of the proximal esophagus leads to the esophageal pouch sign, which is the characteristic ultrasound manifestation of esophageal atresia. The esophageal bag sign is located in the midline of the fetal neck and thorax and is usually located behind the trachea, resulting in a pouch-like anechoic area. Kalache et al. (20) divided the location of the blind end of the upper esophagus into a pouch in the neck and a mediastinal pouch, which was collectively referred to as the esophageal pouch sign in this study. This sign can temporarily appear or disappear with the swallowing activity of the fetus. Therefore, when esophageal atresia is suspected, dynamic observation and multiple and repeated scans of the fetal neck and thorax should be performed. The pouch sign of the esophagus can be observed as early as 22 weeks of gestation (21). In our study, the earliest observation of the pouch sign was at 24 weeks of gestation. The detection rate of the pouch sign increases with the progression of pregnancy and peaks in the third trimester of pregnancy (21). Observation of this direct sign greatly improves the diagnostic rate of fetal esophageal atresia.

If the fetus does not swallow amniotic fluid during the examination, the display rate of the pouch sign is lower (20). In this case, the presence of an esophageal interruption sign can supplement the diagnosis. In recent years, with the upgrades in ultrasound diagnostic equipment and improvements in operator levels, the display of the fetal esophagus and trachea has continuously advanced to the first trimester (18). Chen et al. (22) reported that observing the esophagus using the four-cavity heart view and the three-vessel tracheal view could effectively screen for fetal esophageal atresia, with a sensitivity of 100%, a specificity of 93.6%, a positive predictive value of 6.9%, and a negative predictive value of 100%. An ultrasound image of a normal esophagus revealed a hyperechoic tubular structure; the tube wall presented two or more parallel hyperechoic bands in the longitudinal section and circular hyperechoic bands in the cross section. The size of the lumen changes with the intermittent swallowing of amniotic fluid by the fetus. The esophageal interruption sign manifested as multilayer linear strong echo interruption of the tube wall. The examiners dynamically observed the esophagus in the 13 confirmed patients. When atresia was suspected, the continuity of the esophagus was observed from multiple angles through the short-axis transverse view of the esophagus, the three-vessel tracheal view, and the long-axis view of the paraaortic arch with the rotating probe. According to the final results, 8 patients presented with pouch signs in the upper esophagus, and 4 patients presented with signs of interruption of the esophagus.

Observation of these two direct signs of the esophagus can avoid missed diagnoses. For example, in Patient 4, the amniotic fluid volume and gastric bubbles were normal, but the cervical and thoracic bag sign was found. However, the size could have changed with swallowing, so the diagnosis was EA. Patient 10 was a twin with polyhydramnios, normal gastric vesicles, and fetal FGR. Normal gastric blebs seemed to rule out esophageal atresia. However, FGR is generally accompanied by oligohydramnios. This patient had polyhydramnios, which was not in line with the general characteristics of FGR. Therefore, the examiner continued to explore the fetal digestive tract, neck and thorax and found the esophageal interruption sign, and the fetus was subsequently diagnosed with esophageal atresia. Therefore, it is particularly important to scan the cervical and thoracic esophagus when the two common indirect signs of amniotic fluid and gastric bubbles are normal. Four among thirteen fetuses were confirmed to have growth restriction in pregnancy, with an abdominal circumference <10%. There was 1 neonate with a weight less than 2,000 g and 6 neonates with a weight ranging from 2,000 to 2,500 g after birth. Esophageal obstruction prevents the fetus from swallowing amniotic fluid normally, and the daily protein intake during the second and third trimesters of pregnancy is 2 g less than that of the normal fetus, thus affecting growth and development (23). Therefore, growth restriction can also be considered an indirect sign of EA. When growth restriction is accompanied by polyhydramnios, the digestive tract should be carefully explored, with special attention to the esophagus to improve the diagnostic accuracy.

The consistency rate of the EA classification depended on the observation of the esophagus and tracheal fistula opening and the measurement of the distance between the stumped end of the esophagus. The fetal trachea was located in front of the esophagus, and the ultrasound manifestation was tubular and anechoic. In the coronal section of the trachea, the main trachea and branches on both sides were evident. In the study by Kalache et al. (24), at approximately 15 weeks of gestation, a normal trachea was clear on ultrasound. It has also been reported (25) that the tracheal display rate of 16–36-week-old fetuses was 82%. When combined with an esophagotracheal fistula, the coronal view of the trachea could reveal a fistula opening communicating with the esophagus at or above the bifurcation of the bronchus. In the EA classification, all esophagotracheal fistulas were type II, III, IV or V. Among them, type III has the highest incidence and should be the focus. Moreover, the distance between the upper and lower blind ends of the esophagus should be measured to distinguish types IIIA and IIIB.

In this study, 2 patients with type IIIA EA were misdiagnosed with type IIIB EA. The possible reasons are as follows: (1) After the fetus swallows amniotic fluid, the upper segment of the esophagus fills, and the position of the blind end is lower than that without amniotic fluid filling. (2) When the distance to the blind end of the esophagus was measured during surgery, the patient was under anesthesia, while the intrafetal ultrasound measurement was in the natural state, the blind end was active, and the measurement deviated. Therefore, during the examination, the image should be magnified, and the probe should be rotated at multiple angles to display the anatomical structure of the esophagus and trachea and the relationship between the two. With the cooperation of the fetus swallowing amniotic fluid, the distance from the blind end could be measured and averaged several times to find the fistula openings of the two, and the observation should be extended if necessary.

In this study, in one case of type IIIB that was missed, the two direct signs of polyhydramnios, small/nongastric vesicles, and the esophagus, were not detected on prenatal ultrasound. This difference is considered to be that in type IIIB EA, there is a fistula opening in the lower segment of the esophagus and trachea, and the amniotic fluid can enter the lower esophagus and stomach through the fistula; thus, it manifests as normal gastric and amniotic fluid. Therefore, the gastric bubbles and amniotic fluid were normal. The upper segment of the esophagus and the blind end as well as the communication between the esophagus and the trachea were also observed. It has been reported (16) that 35.7% of cases of EA with distal fistulas have no abnormal ultrasound signs before delivery. Owing to the sample size, the missed diagnosis rate was only 1 case. In addition, for type IV and type V EA, exploration is more difficult because there is no blind end of the upper esophagus and there is an esophagotracheal fistula. If there are no indirect signs, such as polyhydramnios fluid or small gastric bubbles, the diagnosis is easily missed.

In summary, the present study proposes a “three-step method” of prenatal ultrasound examination for the diagnosis and classification of fetal esophageal atresia. (1) Initial screening revealed the following: polyhydramnios + abnormal gastric blebs; (2) confirmation was evidenced with the esophageal pouch sign/discontinuation sign; and 3. classification was performed as fistula positioning + measurement of the distance between the blind ends. The first two steps can be used for the basic diagnosis, which, together with the third step, can be used to determine the degree and accurate classification. The three-step method can improve the prenatal ultrasound diagnostic rate and classification accuracy of esophageal atresia, which can facilitate the provision of individualized diagnosis and treatment programs for postpartum diagnosis and treatment, increase the perinatal treatment rate, and improve the prognosis of pediatric patients.

In approximately 31.6–70.0% of EA cases, EA is associated with other congenital malformations, of which approximately 10% are VACTERL syndrome (26). This study included 13 patients, among whom 6 had multiple malformations, and 1 patient had VACTERL syndrome, which was basically consistent with previous reports. Approximately 6.6% of fetuses with esophageal atresia have chromosomal abnormalities, including trisomy 13 and trisomy 18, and the risk of Down syndrome in EA patients is 30 times greater than that in normal individuals (27). In this study of 13 patients, 12 underwent amniocentesis, and 2 of them had trisomy 18 accompanied by other malformations. The 2 cases of EA were accompanied by other malformations and chromosomal abnormalities, and the outcomes were both induction of labor. Therefore, prenatal ultrasound diagnosis can provide substantial guidance for genetic counseling and eugenics.

In this study, of the 11 fetuses that received surgical treatment after birth, 9 had good postoperative results, and 2 had treatment discontinuation due to the presence of multiple postoperative complications. The distance between the two blind ends of the esophagus and the presence of an esophagus-tracheal fistula are determining factors with respect to the surgical method used after birth. Under normal circumstances, for long-segment esophageal atresia, staged surgery, which is highly complicated, is usually used (28). For example, in that study, for 1 case of type I EA (long segment type), the broken end was relatively far away. Gastrectomy was performed in stage I, the proximal and distal ends of the esophagus were lengthened using the internal stress of the bougie, and the esophagus was fully extended with its own esophageal tissue. When the distance decreased, the blind end of the esophagus was closed using magnetic anastomosis in stage II. For another example, in this study, in a case of type IIIB EA (short-segment type), the two blind ends were close to each other. The fistula tract was ligated and anastomosed with the esophagus via the one-time thoracoscopic approach. The patient was followed after surgery, was able to consume milk by mouth and was growing and developing well.

This study has several limitations. First, its single-center cohort (*n* = 13 confirmed EA cases) and the small sample size reduced statistical power for subtype analysis (e.g., only 1 type IV case). Second, prenatal EA typing relied heavily on operator skill and fetal cooperation during ultrasound, potentially affecting blind-end distance measurements. Future multi-center studies with larger cohorts are needed to validate our proposed ‘three-step method’ and future studies can incorporate microCT for 3D reconstruction of autopsy specimens to validate prenatal findings.

## 5 Conclusion

Esophageal atresia is a treatable malformation with a good prognosis. Prenatal ultrasound examination is an effective method for the diagnosis of esophageal atresia. During the examination, a multiangle and multiview continuous dynamic scan of the blind end of the esophagus is needed to find the fistula opening between the esophagus and trachea, and the distance to the blind end is measured for the diagnosis and clear classification of fetal esophageal atresia. This approach is highly valuable and is significant for prenatal consultation and postpartum surgical plan development.

## Data Availability

The original contributions presented in the study are included in the article/supplementary material, further inquiries can be directed to the corresponding author.

## References

[1] Kassif E Elkan Miller T Tsur A Trozky Y Gur T de Castro H. Dynamic esophageal patency assessment: an effective method for prenatally diagnosing esophageal atresia. Am J Obstet Gynecol. (2021) 225:674.e1–674.e12. doi: 10.1016/j.ajog.2021.06.061, PMID: 34146530

[2] Kuang H Cao H Wang S Luo Y Gao Y Yan L. New ultrasound features in diagnosing fetal anal atresia: a multicenter prospective cohort study. Sci Rep. (2024) 14:22821. doi: 10.1038/s41598-024-73524-3, PMID: 39354020 PMC11445417

[3] Doval L Rousseau V Irtan S. Combined esophageal and duodenal atresia: a review of the literature from 1950 to 2020. Arch Pediatr. (2023) 30:420–6. doi: 10.1016/j.arcped.2023.05.004, PMID: 37328325

[4] Shi X Yang D Cong L Zhong Z Huan X Feng X. The diagnostic value of prenatal ultrasound combined with amniotic fluid AFP and GGTP level detection for congenital esophageal atresia in fetuses. Chin J Anat Clin Surg. (2024) 29:486–91. doi: 10.3760/cma.j.cn101202-20230915-00079

[5] Pardy C D'Antonio F Khalil A Giuliani S. Prenatal detection of esophageal atresia: a systematic review and meta-analysis. Acta Obstet Gynecol Scand. (2019) 98:689–99. doi: 10.1111/aogs.13536, PMID: 30659586

[6] Weissbach T Kushnir A Haber Kaptsenel E Leibovitch L Bilik R Shinhar D. Oesophageal atresia: sonographic signs may prenatally predict surgical complexity. Arch Dis Child Fetal Neonatal Ed. (2022) 107:206–10. doi: 10.1136/archdischild-2021-321836, PMID: 34321245

[7] Wolfe C Jnah A. Tracheoesophageal fistula with esophageal atresia: a case series. Neonatal Netw. (2024) 43:65–75. doi: 10.1891/NN-2023-0051, PMID: 38599775

[8] Weissbach T Kushnir A Yousefi S Massarwa A Leibovitch L Frank DD. The prenatal detection of distal tracheoesophageal fistulas in fetuses diagnosed with esophageal atresia. Am J Obstet Gynecol. (2022) 227:897.e1–9. doi: 10.1016/j.ajog.2022.06.065, PMID: 35940225

[9] Wang C Ning X Duan Y Zhang Z Wang S. Diagnostic accuracy of ultrasonography for the prenatal diagnosis of esophageal atresia and tracheoesophageal fistula. Exp Ther Med. (2021) 21:643. doi: 10.3892/etm.2021.10075, PMID: 33968174 PMC8097193

[10] Zhang J. Jinzhe Zhang 's pediatric surgery. Beijing, China: People's Medical Publishing House (2013).

[11] van Lennep M Singendonk MMJ Dall'Oglio L Gottrand F Krishnan U Terheggen-Lagro S. Oesophageal atresia. Nat Rev Dis Primers. (2019) 5:26. doi: 10.1038/s41572-019-0077-0 31000707

[12] Feng W. Diagnostic value of prenatal ultrasound parameters and esophageal signs in pouch and lower thoracic segment in fetuses with esophageal atresia. Comput Math Methods Med. (2021) 2021:8107461. doi: 10.1155/2021/8107461 34976113 PMC8716201

[13] Ioannides AS Henderson DJ Spitz L Copp AJ. Role of Sonic hedgehog in the development of the trachea and oesophagus. J Pediatr Surg. (2003) 38:29–36. doi: 10.1053/jpsu.2003.50005, PMID: 12592614

[14] Sfeir R Rousseau V Bonnard A Gelas T Aumar M Panait N. Risk factors of early mortality and morbidity in esophageal atresia with distal tracheoesophageal fistula: a population-based cohort study. J Pediatr. (2021) 234:99–105.e1. doi: 10.1016/j.jpeds.2021.02.064, PMID: 33667507

[15] González-Hernández J Lugo-Vicente H. Esophageal atresia: new guidelines in management. Bol Asoc Med P R. (2010) 102:33–8. 20853572

[16] Lepee A Massardier J Atallah A Massoud M Pettazzoni M Huissoud C. Value of biochemical amniotic fluid analysis and fetal magnetic resonance imaging in the prenatal diagnosis of congenital Microgastria. Fetal Diagn Ther. (2024) 51:486–92. doi: 10.1159/000539888, PMID: 38934150

[17] Aoki H Miyazaki O Irahara S Okamoto R Tsutsumi Y Miyasaka M. Value of parametric indexes to identify tracheal atresia with or without fistula on fetal magnetic resonance imaging. Pediatr Radiol. (2021) 51:2027–37. doi: 10.1007/s00247-021-05092-x, PMID: 33988754

[18] Brantberg A Blaas HG Haugen SE Eik-Nes SH. Esophageal obstruction-prenatal detection rate and outcome. Ultrasound Obstet Gynecol. (2007) 30:180–7. doi: 10.1002/uog.4056 17625804

[19] Lu D Chen X Chen C. Discussion on prenatal ultrasound diagnosis of fetal esophageal atresia. Chin J Med Imaging Technol. (2008) 24:718–20. doi: 10.3321/j.issn:1003-3289.2008.05.022

[20] Kalache KD Wauer R Mau H Chaoui R Bollmann R. Prognostic significance of the pouch sign in fetuses with prenatally diagnosed esophageal atresia. Am J Obstet Gynecol. (2000) 182:978–81. doi: 10.1016/s0002-9378(00)70357-7, PMID: 10764484

[21] Kassif E Weissbach T Kushnir A Shust-Barequet S Elkan-Miller T Mazkereth R. Esophageal atresia and tracheoesophageal fistula: prenatal sonographic manifestation from early to late pregnancy. Ultrasound Obstet Gynecol. (2021) 58:92–8. doi: 10.1002/uog.22050, PMID: 32304613

[22] Chen X Sun K Zhou N. Research on the value of prenatal ultrasound four-chamber heart views and three-vessel tracheal view in screening fetal esophageal atresia. Chin J Clin Med Imaging. (2019) 30:5. doi: 10.12117/jccmi.2019.05.010

[23] Dai L Ma H Huang W. Research progress on imaging examination of congenital esophageal atresia and tracheoesophageal fistula. Shandong Med J. (2022) 62:4. doi: 10.3969/j.issn.1002-266X.2022.13.023

[24] Kalache KD Franz M Chaoui R Bollmann R. Ultrasound measurements of the diameter of the fetal trachea, larynx and pharynx throughout gestation applicability to prenatal diagnosis of obstructive anomalies of the upper respiratory-digestive tract. Prenat Diagn. (1999) 19:211–8. doi: 10.1002/(sici)1097-0223(199903)19:3<211::aid-pd487>3.0.co;2-9, PMID: 10210118

[25] Tao Y Li K Chu H Xiao W Li F Jian H. Prenatal ultrasound examination of the diameters of the fetal throat and trachea. Chin J Med Ultrasound. (2009) 2:4. doi: 10.3969/j.issn.1672-6448.2009.02.016

[26] Bradshaw CJ Thakkar H Knutzen L Marsh R Pacilli M Impey L. Accuracy of prenatal detection of tracheoesophageal fistula and oesophageal atresia. J Pediatr Surg. (2016) 51:1268–72. doi: 10.1016/j.jpedsurg.2016.02.001 26932255

[27] Qin L Zhang M Wu T Hai L Jie H Sheng F. The application value of bedside ultrasound in the diagnosis of esophageal atresia in neonates. Chin J Ultrasound Med. (2020) 36:365–8. doi: 10.3969/j.issn.1002-0101.2020.04.025

[28] Kamran A Smithers CJ Mohammed S Izadi S Demehri FR Shieh HF. Management strategies and outcomes of distal congenital esophageal structures in the setting of long-gap esophageal atresia. J Pediatr Surg. (2024) 59:161671. doi: 10.1016/j.jpedsurg.2024.08.011 39209685

